# Laying the Corner-Stone of Rush Medical College

**Published:** 1867-08

**Authors:** 


### SUPPLEMENT.

### RUSH MEDICAL COLLEGE.

### LAYING THE CORNER-STONE—IMPRESSIVE MASONIC CEREMONIES—ORATION BY PAST GRAND MASTER J. ADAMS ALLEN.

Monday afternoon, May 27th, at four o'clock, the corner-stone of the new building for the Rush Medical College, corner of Dearborn and Indiana streets, was duly laid amid most impressive Masonic ceremonies. Representatives of the Apollo and Chicago Commanderies of the Knights Templar were present, and also the Masters and Wardens of most of the Chicago Masonic Lodges. The Grand Lodge was fully represented.

Prayer was offered by the Worthy Grand Chaplain, Rev. O. H. Tiffany.

The exercises were inaugurated by Worshipful Master, J. W. Clyde, of Oriental Lodge, who briefly explained the objects of the meeting.

The appropriate officers then, with their jewels of office, tried the stone, and reported it to be correctly formed, the workmen having properly performed their labors.

Oil, wine, and wheat was next scattered upon the stone, when several articles were placed in the cavity and the work formally concluded.

#### CONTENTS OF THE CORNER-STONE.

The following is a list of the articles placed in the copper box, deposited in the cavity beneath the corner-stone:

Charter of the College.

Names of the present Board of Trustees, the Faculty, and Alumni.

Epitome of College history.

Description of new building, and names of architect and contractors.

Code of medical ethics.

Constitution and by-laws of Illinois State Medical Society.

Twelfth annual report of the Chicago Board of Education.

Last annual report of the Board of Trade.

Daily papers of Chicago.

Regulations, etc., of the Marine Hospital.

Reports of St. Luke's Hospital, Chicago Eye and Ear Infirmary, and Hospital for Women and Children.

Board of Health papers.

Chicago *Medical Journal.*

*American Journal of Medical Sciences.*

Chicago *Medical Examiner.*

Pamphlet copy of the *Catholic World.*

Fractional currency and Federal coins.

Last City Directory, (Bailey's.)

Tourists' Guide of City of Chicago, (Goodsell's.)

Photograph and Biography of Dr. Brainard, deceased.

Photographs of the Faculty of the College.

Photograph of W. W. Boyington, Architect.

Upon the conclusion of the ceremony of laying the corner-stone, the Worshipful Master introduced to the assemblage

HON. WILLIAM B. OGDEN.

Mr. Ogden said, that in the absence of the President of the College, he appeared as Chairman of the Board of Trustees to thank the Masons for their performance of the ceremonial duties of the occasion, and the people for their attendance. In a pleasing manner he spoke of the rapid growth of the city during his residence here. Thirty-two years ago he had felled timber near the very site they were now occupying; then the red man's wigwam could be seen in the city; now the forests had disappeared, the red man had gone away, and institutions of wealth and learning could be seen on every hand. The citizens of Chicago have much to be grateful for and proud of.

In conclusion, Mr. Ogden introduced Prof. DeLaskie Miller, who spoke as follows:

### HISTORY OF THE COLLEGE.

Fellow-citizens: We have assembled at this place this afternoon, not to inaugurate an entirely new enterprise, or to reanimate an unsuccessful experiment, but to witness an interesting ceremony connected with the prosperity of one of the noblest institutions in the land—the laying of the corner-stone of a new edifice, which has become indispensible by the requirements of this city and the vast territory tributary to it.

That this work has become a necessity, which may not be deferred, a few facts will fully substantiate.

The charter of Rush Medical College was obtained in the year 1837. Soon thereafter a Board of Trustees was appointed, who, without much delay, organized a Faculty, fully authorized to teach the science of medicine in all its branches, and confer the Degree of Doctor of Medicine.

The first complete course of lectures was not given, however, till 1843, and then to a class of 22. From that time to the present, full courses of lectures have been given annually, without abatement in any particular, and the last to a class numbering nearly 300. In the grand aggregate, some thousands of yonng men, seeking to qualify themselves to meet the responsibilities incident to our beneficent profession, have sought instruction here. Of this number about 1,000 have received the honors of the institution, and carry with them not only our diploma—the evidence of their attainments—but, better still, the knowledge and ability to cope successfully with disease and medical error in all its specious forms.

Soon after the reörganization of the Faculty—about seven years ago—it became apparent that the accommodations afforded by the old College building would soon become inadequate; and this apprehension has been replaced by demonstration. For during the last three years, the size of the class has been limited only by the capacity of the building, and for this reason numbers have patronized other institutions, who greatly preferred this College, proving that this is no chimerical scheme.

It is the united purpose of those immediately interested in this enterprise, and responsible for its success, that in the future, as in the past, the course of Rush Medical College shall be characterized by advancement in all the improvements drawn from the lights of true science; and with our motto—"Excelsior"—our march shall be onward and upward.

I now have the pleasure of introducing Prof. J. Adams Allen, the orator of this occasion.

Dr. Allen then delivered the following

ORATION, which was listened to throughout with the greatest attention, notwithstanding the fact that the rain was falling steadily all the time:

*Officers and Brothers:* It is fitting, it is right, that the chief foundation stone of this new structure should be laid with the sober but impressive pomp of ancient Masonic ceremonial.

Architecture, which is but a branch of Masonry, can, like the latter, be considered both as operative and speculative; for the operative completion of the structure but fixes in solid material shape the speculative building, which before existed in all its parts in as distinct perfection, in the mind of the architect.

All the forms of art, indeed, are but the embodiment in material shape of the inner, unseen thought of the artist. Nay, by a higher essay of the truly philosophic mind, it is to be seen that the great globe itself and the universe of worlds are but the visual manifestations of the wondrous idea of the Supreme Architect, expressing itself, (with deep reverence be it spoken,) as one has said, artistically in space.

We turn our eyes upon this our globe, and study its overlying strata stamped with the effigies of dead races of animal and vegetable life—upon its surface, life in action in all its myriad varieties, the ceaseless operation of forces which, as moment succeeds moment, and age follows upon age, by strange paradox, prove uniformity of action, by constancy of mutation. Then we turn the telescope to the far off heavens, and with the waves of light come whisperings from those distant worlds of the wisdom and power of Him who holds the circling spheres in the hollow of His hand, and commands the laws to which all, whether animate or inanimate, material or immaterial, are in eternal subjection. Out of the silence comes the "still small voice," and nature in repose speaks trumpet-tongued to the hearing ear and the understanding heart. As the philosopher wrote of motion: "Even without change of place it has its force; and bodies big with motion labor to move but stir not, whilst they express an energy beyond our comprehension."

It is fitting, therefore, upon an occasion like this, when we celebrate the laying of this corner-stone amid masses of solid work of operative masonry, to ponder well the thoughts which are to be expressed by this new edifice.

The architect here calls these mineral masses to give body to his thought of fitness, of strength, of durability, of majesty, of proportion, of beauty. Nor doubt we that these shall each find expression around and above this corner-stone—for even now the aid of the pencil has given them visual form—those thoughts even now are immortal, though *he* pass away forever before stone rises upon stone to the last necessary sound of the gavel.

But in this corner-stone we find other thoughts than those of the architect. The work of the operative Mason in shaping this well-squared ashlar from the native rough—a material protest against the rude natural state—is paralleled in speculative Masonry, which at this time, and during all times, is and has been a living protest against savagery, brute force, and especially *ignorance*, which makes that force a thing to be feared. And thus this corner-stone becomes a mile-stone along the route of human progress. Those who have passed beyond, place it here for those who come after.

It has ever been the pride of Masonry, that even during ruder ages, when society was comparatively chaotic, and property and life but the playthings of the strongest arm, its potent influence was always in favor of the "humanities,"—it softened manners and robbed customs of their ferocity. While it laid broad and deep the foundations of castles and cathedrals, of palaces and churches, it also fostered, by its benign influence, the liberal arts and sciences, preserving at least their germs among its arcana, even amid the havoc of revolutions, and the corroding rust of otherwise benighted centuries. And it was this peculiarity which distinguished our ancient craftsmen from the other architects of antiquity. Mere piling of stone upon stone, although deftly arranged, and curiously carved, does not satisfy the Masonic thought. The grandeur of the Coliseum at Rome, with its vast amphitheatric tiers rising in sullen majesty, so that tens of thousands at once could look down upon its central arena, or upon the throne of Vespasian, its proud builder, remains to us but the monument of a barbarous people, when we recollect that in that arena only wild beasts fought, or more savage gladiators were butchered, to make a Roman holiday.

"Ad cædes hominum prisca amphitheatra patebant;
Ut longum discant vivere nostra patent."

Around and above this "WELL FORMED, TRUE, AND TRUSTY" corner-stone, will arise another amphitheatre, devoted to the science and art of prolongng life, and crowning it with its necessary happiness—health.

Time was when individual life was deemed of but little value, for the objects to be gained by its continuance were of little importance. The war or pestilence which thinned the crowded city was looked upon by its rulers as a relief from threatened annoyance. "The Romans," says Plutarch, "always had recourse to war, like good physicians, to expel dangerous humors from the body politic." The pestilence was but a natural outlet for the same.

But in the transition from pristine and mediæval semi-barbarism to the present era, there has been a concurrent advance in the intrinsic value of individual life. *Intrinsic*, for the objects and uses of that life have become more numerous, more important, and more immediately interwoven with the general welfare. As in numbers the integer, though but a unit, expresses not only its own value, but, by position, the relative value of the figures with which it is written, so, the individual is now by no means isolated, but of a use and importance not to be estimated without reference to the entire body of society. The life of the soldier rises in importance with the interests at stake in the war. The life of the ruler is not a mere personal interest, but may involve the very existence of law and order. The life of the farmer, the manufacturer, the merchant, the artisan, the professional man—each is to be considered, not solely from the numbers engaged in these several pursuits, but from their relation to the general weal. The sum total characterizes the age, illustrates its tendency, and is its part of universal history. Thus intimately are the parts knit and connected in the harmonious frame-work of society. The loss of health or life robs society of that service which would otherwise accrue, whence results gereral loss. The recognition of this fact has been formally made known in the care exercised by Government over armies and navies. Not only have they been provided with the ordinary necessaries of life, but, moreover, extreme pains have been taken that they should be defended against the attacks of disease, which might at once destroy their efficiency. This has been sought by the training and appointment of competent medical attendants. At the present day there is not an army or navy, belonging to any civilized Government, without its regularly appointed corps of scientific medical officers. To facilitate this object still further, most of the prominent Governments have taken care to establish and favor with a fostering influence institutions where the elements of the art preservative of life could be acquired, and hospitals where the highest perfection of its practice could be illustrated. The General Government of this country have so far acknowledged the necessity of this course that they will not admit to the medical corps of either army or navy any but those who have attained a high grade of acquirement in medical science. During the unhappy struggle from which the nation has just emerged, the wisdom of this course, and the great strides in advance which have been made in medical art, have been most notably illustrated by a freedom from disease and mortality heretofore unparalleled in the annals of war.

Thus, by the constituted authorities of all Christendom, it is declared that the preservation of the lives of such of their citizens as are employed in military or naval affairs, is an object of national concern, and should be solicitously regarded.

We might go further than this, and lay it down as an incontrovertible principle, that the citizen in civil life is also entitled to similar care. As men, and not money, are the real sinews of war, so men, and not machinery, are the sinews of peace. While the nation, by its arms of offence and defence, is making itself felt abroad, by its Briarean operatives at home it is, or should be, building up the more permanent framework of moral, intellectual, and material power. The life which lost in the army involves but a temporary deficiency, lost in the ranks of operative effort casts hundred-fold the shadow. The normal condition of society is peace; its unnatural or diseased state is war. War is an accident of external relations; peace is accordance with the law of its existence. It is fitting, therefore, that every well organized Government should scrupulously provide, whether during peace or war, for the preservation of the first of rights to its citizens—the right of life, and its corollary, health. The position is one which contravenes argument, and needs to be but clearly stated to command admission.

Beyond even this, as every individual is to a certain extent the property of the nation, he has no right to sacrifice his own life or health, either directly by his own act or indirectly by maluse of the means for their preservation, no more than to mutilate himself or commit suicide. Common sense and the common law, which should be its mirror, indorse the proposition as tenable. How far this abstract truth should be carried into practical results may be a matter for debate, but the clearly derived inference from this and the preceding propositions is, that the sovereign power of every State is bound by every precept of right, justice, and sound policy to provide its citizens with the best of known means for the preservation of life and the continuance of health.

Unfortunately the peculiar political ideas in ascendancy in this country, from the first organization of the Government down to the present time, have prevented practical acknowledgment of this prime duty, save in rare instances, and even in these to the most limited extent. Government has meddled with (and not unfrequently marred) almost every conceivable interest but this, which has been left wholly to private enterprise. It has recognized, often liberally, the claims of educational institutions, from the common school to the University, which is well, but it has left uncared for the physical frame of the young and the old alike—so that the former bring small aid to the operative energy of the mass, and the latter fill untimely graves.

The modicum of education afforded in the primary schools is not so immediately designed for the benefit of the individual as for that of society. The public authority provides the means of instruction for public ends. It is better to build school-houses than jails—to pay school-masters than prison wardens and guards.

The same principle holds true with reference to the University course. The high degree of education imparted to the few at the expense of the many, is looked upon by the narrow-minded as an unjust partiality, which is far from the truth. The few, though incidentally individually benefitted, are not thus favored for this object. The grand underlying reason is that knowledge is like water, constantly tending to diffuse itself; and the experience of the world shows that the educated mind constantly tends to awaken the energies, enlarge the perceptions, and correct the waywardness of uneducated mind. An ever widening circle of valuable influence surrounds the former, of which the latter is immediately and largely participant. This is the object sought, and thus it is attained.

The building to be erected around and above this corner-stone is to be devoted to one branch of this higher education. It happens that the individuals who will attend upon the courses of instruction herein to be given will, afterward, make use of their acquirements in the very practical and very prosaic business of getting their daily bread, and in rare, and perhaps fabulous, instances, in securing a credit side to their banker's book. Were this all, it would scarcely seem worth while to call public attention to what would in such case be a mere private enterprise. But those who have this institution under their charge, and by whose personal efforts and sacrifices this structure is to gain completion, look upon their relations to society with a different eye.

In this age of the world, that which is necessary to be done *is* done, even though not by those whose clear duty it is to perform the work. If Government persists in playing the part of Hercules, and will not come when prayed for to put its broad shoulders to the wheels of progress, the car must move, any way—the wagoners are uot content to be perpetually in the mire. Wo be to the unhappy wight, who, thinking otherwise, would block the forward wheels!

A prouder temple than those of olden time erected to Æsculapius and Hygeia, is here to arise, for it will be dedicated to *truth* and *humanity*. Herein the diligent searcher after the true shall be enlightened and increase his lore, while the lover of his fellow man shall find means to alleviate suffering, and cheer with ready sympathy those whose unfortunate lot it is to fall under the sway of disease.

It is not arrogating too much to say that the condition of the science of medicine, in any particular age of the world, is an accurate reflex of the general science of that period. It is the barometer of the time, indicative of the atmosphere of knowledge. If the absurdities and excrescences which have marred its history at times, elicit ridicule and demand contempt, we of the profession are comforted by the reflection, warranted by history, that it is impossible to say anything so absurd that it has not at some period been advanced and defended by philosophers.

Mr. Weller pithily and sententiously remarks, "There is a great deal of human nature among men—especially widders," and we may be pardoned for assuming the same general statement, adding—especially doctors. The devious ways through which medical knowledge has been sought—the stumbling footsteps even of its most earnest students, would be sufficient food for satire, were it not for the primal fact that thus is it ever in the search for truth. Poor human nature always, even in progression, has to tack and veer against the head winds of prejudice and ignorance. "The way of truth," says Milton, "is plainness and brightness; the darkness and confusion are all our own."

Another great trouble is, that a little truth wrongly interpreted, becomes at once more dangerous than an absolute falsehood. Put a little fragment of truth under a wrong-headed man, and "he gets up into clamor over it as a lame man gets on horseback," to take a beggar's ride—with what kind of result the proverb makes us sufficiently familiar.

An ignorant age is superstitious and credulous, and an ignorant man believes, "at sight," the baldest delusions to be Gospel verity. If he has been baptized *doctor*, the ordinance unfortunately does not help the matter. Starting from his little point of truth as a center, he revolves about it at the most magnificent distances, or flies away, in tangential fashion, nevertheless very humanly. The professional errors of one age become the popular doctrines of the next. Thus, the sins of Æsculapian fathers are visited upon other people's children, to the immense discomfort of a whole generation.

Unhappily, this does not exhaust the capacities for error which exist among doctors and other people. There are numerous and pestilent follies and absurdities, of which so-called educated minds only are capable. Politicians are very fond of speaking about "educating the people up" to their peculiar views—(usually erroneous and pestiferous.) The human nature among doctors here shines out from the darkness of its surroundings. A long course of education may bring any man, even a doctor, to accept absurd views with religious faith, just as the physical system may be "educated up" to the endurance of opium, of whiskey, arsenic, or stump speeches. Prejudice, which Burke says renders a man's virtue his habit, unluckily makes his vicious notion a shirt of Nessus. "Men are wonderfully happy," says Lord Shaftesbury, "in a faculty of deceiving themselves, whenever they set heartily about it. A very small foundation of any passion will not only serve us to act it well, but even to work ourselves in it beyond our own reach."

Education is a developement of the faculties, whether mental or physical, and some people think education is the end of the matter. But the faculties may be developed mischievously—the mental athlete may become a bully or a prize-fighter, or defend vigorously the wrong idea which has come uppermost in his hypertrophied ignorance. Education is not wisdom by any manner of means. Tamerlane had power enough when he piled up a pyramid from the skulls of his victims. Wisdom would have taught him rather to build factories and cities, iron-clad Dunderbergs, steam engines, and paper money. He had better have organized Oriental "Boards of Health," with all risk of having them declared unconstitutional afterward.

Education detects the flaw in language; wisdom recognizes the noble intent, and carries out the grand design.

Education fills hungry graves with precocious children, and shortens the span of life of the Herculean gymnast. Wisdom seeks symmetrical developement, both of mind and body—believes that water cannot rise higher than its fountain head, or power be created from nothing, and therefore secures a base of supplies before it forces the action of mind or muscle.

It is not sufficient to have power—the world is full of that—but to know how properly to apply it. It is not sufficient to have ideas, but there must be understanding, also. It is not alone enough to have learning—there are dunces in abundance whose heads are gorged with that. It is not enough to pore over black-letter volumes by the traditional "midnight oil." The written words, all things which the senses can apprehend, or which memory can call up from the stores of the past, must be subjected to the stern test of an educated wisdom which sharply separates the true from the false—the glitter from the gold. The sunlight must displace the farthing candle—not even the comet or the meteor will do in place thereof.

We have laid this corner stone of a new temple of science, and the priests of that temple fervently believe that in their offerings they will not imitate the Athenians, who, the Apostle tells us, put their sacrifices on an altar inscribed, "To the unknown God." They expect to obey the injunction:

"Trust not the frantic or mysterious guide,
Nor stoop a captive to the school man's pride;
On nature's wonders fix alone thy zeal,
They dim not reason."

Very little, comparatively, of the teachings within the walls here to arise will admit of controversy. As the wars which attract the world's attention are oftener about points of etiquette or fractional lines of boundary, than about the "eminent domain" of the realm, so the acrimonious disputes and clangoring debates for which the profession is unpleasantly famous, and over which the wits make themselves merry, are rather the ritualistic questions than the nine and thirty articles. The affairs in dispute are but the hem of the robes of Æsculapius. Nevertheless the contention has its use, and the satire fulfills its mission.

A good healthy skepticism on the one side, and sharp laughter on the other, are counter-irritants, likely to wake up action in the most pachydermatous mind. An easy-going intellect is as apt to be filled up with rubbish as a State Church is to become corrupt, a party without opposition to degenerate, or an idle man to get dyspepsia. *Per aspera ad astra.* Jacob's second wife, who cost him fourteen years' hard service, was, probably, as much superior to the seven-year purchase, as Leah herself to the modern damsel, who is won in a day and divorced the next.

The medical profession will improve, and has improved, by attrition, as the diamond in the hands of the lapidary.

The great central idea upon which medical science now stands with confidence is that, in both mind and body, man is subjected to the domination of immutable laws. The tide of his being rises upon the ocean of law. Above, beneath, around, within him, they equally hold sway, and obedience is the condition of his spiritual and physical life.

While knowledge was being slowly and painfully accumulated by our fathers, they groped in a darkness rendered more profound because this primal truth was not developed. "Knowledge comes, but wisdom lingers," when the student merely accumulates facts without gathering them into the links of the grand chain of universal law. As an intelligent writer has said: "The book of nature is a sealed book to his untutored and uninstructed vision. The laws which it contains, each of which he is commanded to obey, under great and severe penalties for disobedience, or stimulated to obey by the promise of high reward, are written in a language which he does not understand, and which is only to be comprehended by the labor of observation and study." In observations and studies of this kind, it has come to pass that we find man has relationships and affinities with all that lives, or has lived. He is not solitary; and if we inquire into the arcana of all nature, the every answer will tend to explain more and more fully his peculiar nature. His relationship to other created beings is not merely general, but minute and all-pervading. To understand his animal mechanism, or mental part, it is imperative that we become acquainted with all else that has life, and with all those phases of matter and force which, from hour to hour, impress constant changes upon him.

Hence the science of his being becomes universal science. There is no knowledge but what bears upon his destiny; there is no law but whose orbit has him for a center.

Surely it is no unimportant work in which we are this day engaged, for above this corner-stone will this study be made the tribute to a greater than imperial Cæsar.

The individual man, though a Cæsar even, is little noteworthy. If he sinks under disease and death, the great ocean of life closes over him with scarce a ripple on its surface. But man united with his fellow-man can control even the primal forces of nature to work his will. The great laws embrace not the Emperor only, but the human race.

Out of the atmosphere of hoarse disputes is gradually emerging a knowledge of the great laws of man's being—of life and health. We, who are devoting our energies to this great work, and the noble edifice to arise upon this corner-stone consecrated to its uses, shall have but a fleeting existence. But when the teachers have gone to their last resting place, and these walls shall crumble around this corner-stone, there will be other hands to take up the grand work, and build new temples, and achieve more glorious triumphs in the contest with nature, to wrest from it the mysteries of health and life.

And at this word we are saddened at the reflection that he who, a quarter of a century since, founded this College, and but a few short months ago was a leader in the councils of the Faculty with regard to the erection of the building upon whose foundations we are now gathered, has been suddenly torn from our ranks by all-powerful Death. Brainard is dead! His works and achievements are his most enduring monument; their record his proudest eulogium.

We miss, also, to-day, from our number, one who was associated with Brainard from the organization of the institution until he stood by his death-bed, in autumn last. Arduous labors, incident to his connection with the army during the war—duties most conscientiously performed—and zealous devotion to his favorite studies, have broken the power of his physical frame, and he seeks now, by a few weeks relaxation from toil, to recuperate his wearied energies. Safely and speedily, with renewed health and vigor, may he return to the active duties of his position as President of the College, to rejoice the hearts of his colleagues by his genial companionship, and strengthen them by his wise counsels. Live Blaney, the patriot, the gentleman, the Mason, tried and true.

Another of the number, in a few days, leaves us for a few months visit to the Old World. He will lay under contribution those renowned in medical science and letters, and gather from their sources, in those transatlantic fields, whatever will most enrich the College and profession which he loves. His personal presence forbids the eulogium which springs to my lips. On his return he will find the capstone upon the arch—the turret crowning the completed building; but he will find no change in the love and respect which all who know him entertain. May the ocean forget its storms while it carries him; and upon land let accident and disease be far from him. Return to us early, Brother Freer; but *serus in coelum!*

Officers and Brothers, on behalf of the Trustees and Faculty of Rush Medical College, I thank you that you have laid this corner-stone with the solemn rites of the ancient Order. The ceremony itself originated with the ancient craft, but it has been so frequently imitated by other organizations that many have lost sight of its peculiar Masonic significance. This imitation, in itself, is a recognition of the power of that symbolic teaching which is the grand characteristic of Masonry. From the tiled floor of the Lodge to the Grand Orient, this teaching illustrates the pathway of the true Mason.

The fitly laying of the corner-stone, deemed so necessary by operative masons, suggests, by impressive parallel, that the commencement of every work of life should be thoroughly tested by the moral masonic implements—the square, the level, and the plumb, else the superstructure will be liable to fall at touch of the lightest disturbing cause. It is the first step that tells—for it marks the direction. There is a world of wholesome truth in the trite saying: "Be sure that you are right—then go ahead."

Progress is the pride of the time, but it may be toward the nethermost region rather than Heaven.

These walls may rise with rapidity, but unless the operative mason adjusts his lines by those of this "tried and trusty corner-stone," they will fall so much the faster.

The life of Man, to us Masons, is a spiritual temple, built of ashlar, shapened at our own will. We can make it "a beauty and a joy forever," or mar it to utter ruin.

But let the corner-stone of every work be based upon the sure foundation of the moral law; let the cardinal tenets of our profession, Brotherly Love, Relief, and Truth, be applied to it as unerring tests, and the pillars of the temple can never be shaken, nor will the fires upon its altars ever go out.

"Visit the sick; bury the dead; educate the orphan;" these injunctions indicate the kind of work to be done, but not all. They are the necessary parts of that symbolic building of which Universal Benevolence is the chiefest corner-stone.

THE BENEDICTION

was then pronounced by Rev. O. H. Tiffany, D.D., Grand Chaplain, after which the assemblage dispersed.

